# Alcohol Consumption, Risk of Periodontitis and Change of Periodontal Parameters in a Population‐Based Cohort Study

**DOI:** 10.1111/jcpe.14154

**Published:** 2025-03-13

**Authors:** Sebastian‐Edgar Baumeister, Janka Schössow, Gustavo G. Nascimento, Henry Völzke, Thomas Kocher, Birte Holtfreter

**Affiliations:** ^1^ Institute of Health Services Research in Dentistry University of Münster Münster Germany; ^2^ Institute for Community Medicine University Medicine Greifswald Greifswald Germany; ^3^ National Dental Research Institute Singapore, National Dental Centre Singapore Oral Health Academic Clinical Programme, Duke‐NUS Medical School Singapore Singapore; ^4^ Department of Restorative Dentistry, Periodontology, and Endodontology University Medicine Greifswald Greifswald Germany

**Keywords:** alcohol consumption, observational study, periodontitis

## Abstract

**Aim:**

To investigate the association of alcohol consumption with periodontitis risk and change in periodontal parameters over time.

**Methods:**

Using data from 1285 participants of two population cohort studies embedded in the Study of Health in Pomerania, we associated baseline average alcohol consumption with incident periodontitis measured after a median follow‐up time of 5.0 years, adjusting for confounding and selection bias using multivariable regression and multiple imputation.

**Results:**

Baseline alcohol intake was prospectively associated with a higher risk of periodontitis (relative risk of 1.08 (95% confidence interval: 1.06, 1.10) and 1.23 (1.17, 1.28) for 30 and 60 g per day (g/day) versus 10 g/day), deeper periodontal pockets, higher clinical attachment levels (CAL) and a higher proportion of sites with probing depths and CAL ≥ 3 mm and ≥ 4 mm.

**Conclusions:**

Our results support the hypothesis that higher alcohol intake modestly increases the risk of periodontitis. Sensitivity analysis suggested that unmeasured confounding and selection bias could explain the observed association.

## 1 Introduction

Oral health is a multidimensional concept encompassing physical, psychological, emotional and social domains, all integral to overall health and well‐being (Glick et al. 2017). Periodontitis is a chronic inflammatory disease of the tooth‐supporting tissues. In 2021, the global age‐adjusted prevalence of severe periodontitis was 12.5% (Nascimento et al. 2024). Affected patients are at an increased risk of tooth loss, edentulism and masticatory dysfunction, which negatively impact their nutrition and self‐esteem. Periodontitis is linked to several systemic comorbidities (e.g., diabetes, cardiovascular diseases [CVD], rheumatic arthritis) (Botelho et al. 2022). Tobacco smoking and diabetes are causal risk factors for the development and progression of periodontitis (Baumeister et al. 2021; Chapple et al. 2017). The burden of periodontitis has remained unchanged over the past three decades (Nascimento et al. 2024), which raises concerns that current approaches to its prevention and control might be insufficient. The role of alcohol in periodontitis has gained particular attention because alcohol is a potentially modifiable behavioural risk factor. Alcohol consumption is a major risk factor for poor physical and mental health, accounting for about 1.8 million deaths in 2020 and representing the leading risk factor for mortality among males aged 15–49 years (GBD 2020 Alcohol Collaborators 2022). Since the 1990s, alcohol consumption has increased in many low‐ and middle‐income countries. Evidence consistently demonstrates alcohol's hazardous effects on several major diseases, including various types of cancers, CVD, liver cirrhosis, infectious diseases and injuries (WHO 2018).

Estimates of the periodontitis burden attributable to alcohol intake have typically been based on risk estimates derived from observational studies. Several systematic reviews and meta‐analyses have evaluated and quantified the association between alcohol consumption and periodontitis (Oliveira et al. 2022; Pulikkotil et al. 2020; Wang et al. 2016). (Oliveira et al. 2022) analysed evidence from systematic reviews on the association between alcohol consumption and periodontitis risk, supplemented by an updated meta‐analysis of prospective cohort studies. Their pooled results revealed no significant prospective association between alcohol intake and periodontitis risk, except among men in low‐ and high‐middle‐income countries. Using the Risk of Bias in Non‐Randomised Studies of Interventions (ROBINS‐I) tool, the authors assessed bias in the included cohort studies and found that most exhibited a serious risk of bias. Key sources of systematic error included participant selection bias, unadjusted confounding and exposure misclassification.

We conducted a population‐based cohort study in north‐eastern Germany, collecting data on alcohol consumption and performing dental examinations at multiple time points to provide additional observational evidence. We analysed the association between baseline average alcohol volume consumed (termed ‘average alcohol consumption’) and the risk of incident periodontitis. Additionally, we assessed the relationship between baseline alcohol consumption and follow‐up periodontal parameters. Sensitivity analyses were performed to evaluate the potential impact of unmeasured confounding and selection bias on the observed associations.

## 2 Methods

### 2.1 Study Design

The Study of Health in Pomerania (SHIP) project consists of two independent population‐based cohort studies (SHIP‐START and SHIP‐TREND) conducted in north‐eastern Germany (Völzke et al. 2022). SHIP‐START employed a two‐stage stratified cluster design to sample Caucasian individuals residing in the study area. The sampling process initially selected 3 cities, 12 larger towns and 17 surrounding villages. From these, a sample of 7006 subjects, stratified by age and sex, was drawn proportionally from population registers based on municipality size. After excluding 741 neutral losses (126 deaths and 615 migrations), 4307 of the 6265 eligible subjects participated in the baseline examinations (SHIP‐START‐0) between 1997 and 2001, yielding a baseline response rate of 68.8%. Follow‐up examinations (SHIP‐START‐1) were conducted from 2002 to 2006, involving 3300 subjects (83.6% follow‐up response rate), with 130 passive non‐responders due to migration and 231 deceased subjects. For SHIP‐TREND, a stratified random sample of 10,000 adults aged 20–79 years was drawn from population registries, stratified by age, sex and city/county of residence. After excluding 851 migrated and 323 deceased individuals, the net sample comprised 8826 persons. Of these, 4420 subjects were recruited for baseline examinations (SHIP‐TREND‐0) conducted from 2008 to 2012, achieving a baseline response rate of 50.1%. The first follow‐up examination (SHIP‐TREND‐1) took place between 2014 and 2018, with 2507 participants (56.7% follow‐up response rate). A flow chart of the study population is provided in Figure S1. All participants provided written informed consent, and the study was approved by the Ethics Committee of the University of Greifswald.

We combined baseline data from SHIP‐START‐0 and SHIP‐TREND‐0 and conducted prospective analyses using follow‐up periodontal outcomes measured in SHIP‐START‐1 and SHIP‐TREND‐1, respectively. Two analytical datasets were created. The first dataset, designed to study incident periodontitis, excluded individuals with moderate or severe periodontitis at baseline, as defined by the Centers for Disease Control and Prevention/American Academy of Periodontology (CDC/AAP) case definition (Eke et al. 2012). The complete‐case dataset, after inclusion of participants with missing values on baseline periodontitis or covariates, included 1285 participants (Table 1). The second dataset was constructed to examine the association between baseline alcohol consumption and follow‐up periodontal measurements. This dataset retained individuals with moderate or severe periodontitis at baseline but excluded those without follow‐up periodontal measurements or missing baseline covariates, resulting in 3173 study participants (Table 2). Descriptive statistics for both datasets, without listwise deletion, are provided in Tables S1 and S2.

**TABLE 1:** Baseline descriptive statistics of analytical dataset 1 to examine the association between average alcohol consumption and risk of incident periodontitis in SHIP‐START and SHIP‐TREND.

	SHIP‐START‐0	SHIP‐TREND‐0	Total
Sample (complete case dataset), *n*	628 (48.9%)	657 (51.1%)	1285 (100.0%)
Periodontitis			
No or mild	628 (100.0%)	657 (100.0%)	1285 (100.0%)
Moderate or severe	0 (0%)	0 (0%)	0 (0%)
Mean pocket probing depth, mm	2.11 (0.35)	2.21 (0.27)	2.16 (0.32)
Proportion of sites with pocket probing depth ≥ 3 mm	0.32 (0.18)	0.30 (0.16)	0.31 (0.17)
Proportion of sites with pocket probing depth ≥ 4 mm	0.03 (0.04)	0.03 (0.05)	0.03 (0.05)
Mean clinical attachment level, mm	1.44 (0.74)	1.35 (0.61)	1.40 (0.68)
Proportion of sites with clinical attachment level ≥ 3 mm	0.21 (0.19)	0.13 (0.15)	0.17 (0.18)
Proportion of sites with clinical attachment level ≥ 4 mm	0.04 (0.08)	0.03 (0.06)	0.04 (0.07)
Average alcohol consumption, grams of ethanol per day	13.13 (18.10)	8.08 (10.76)	10.55 (15.02)
Male sex	265 (42.2%)	280 (42.6%)	545 (42.4%)
Age, years	43.11 (10.46)	44.95 (10.26)	44.05 (10.40)
Schooling attainment			
< 10 years	101 (16.1%)	42 (6.4%)	143 (11.1%)
10 years	374 (59.6%)	379 (57.7%)	753 (58.6%)
> 10 years	153 (24.4%)	236 (35.9%)	389 (30.3%)
Smoking status			
Never smoker	314 (50.0%)	369 (56.2%)	683 (53.2%)
Former smoker	146 (23.2%)	140 (21.3%)	286 (22.3%)
Current smoker	168 (26.8%)	148 (22.5%)	316 (24.6%)
Pack years	6.46 (10.05)	5.46 (9.11)	5.95 (9.59)
Diabetes	11 (1.4%)	30 (3.4%)	41 (2.5%)

*Note:* Study of Health in Pomerania (SHIP) Entries are means (standard deviations) for continuous variable and numbers of observations (%) for categorical variables.

**TABLE 2:** Baseline descriptive statistics of analytical dataset 2 to examine the association between average alcohol consumption and follow‐up periodontal parameters in SHIP‐START and SHIP‐TREND.

	SHIP‐START‐0	SHIP‐TREND‐0	Total
Sample (complete case dataset), *n*	1723 (54.3%)	1450 (45.7%)	3173 (100.0%)
Periodontitis			
No or mild	635 (37.3%)	659 (45.9%)	1294 (41.2%)
Moderate or severe	1067 (62.7%)	776 (54.1%)	1843 (58.8%)
Mean pocket probing depth, mm	2.52 (0.65)	2.52 (0.56)	2.52 (0.61)
Proportion of sites with pocket probing depth ≥ 3 mm	0.47 (0.23)	0.42 (0.21)	0.44 (0.22)
Proportion of sites with pocket probing depth ≥ 4 mm	0.13 (0.16)	0.13 (0.16)	0.13 (0.16)
Mean clinical attachment level, mm	2.69 (1.56)	2.35 (1.39)	2.53 (1.50)
Proportion of sites with clinical attachment level ≥ 3 mm	0.50 (0.32)	0.39 (0.32)	0.45 (0.33)
Proportion of sites with clinical attachment level ≥ 4 mm	0.28 (0.29)	0.22 (0.27)	0.26 (0.29)
Average alcohol consumption, grams of ethanol per day	13.46 (19.75)	9.14 (13.81)	11.49 (17.42)
Male sex	848 (49.2%)	712 (49.1%)	1560 (49.2%)
Age, years	48.90 (11.67)	50.28 (11.85)	49.53 (11.77)
Schooling attainment			
< 10 years	529 (30.7%)	173 (11.9%)	702 (22.1%)
10 years	856 (49.7%)	825 (56.9%)	1681 (53.0%)
> 10 years	338 (19.6%)	452 (31.2%)	790 (24.9%)
Smoking status			
Never smoker	766 (44.5%)	737 (50.8%)	1503 (47.4%)
Former smoker	471 (27.3%)	379 (26.1%)	850 (26.8%)
Current smoker	486 (28.2%)	334 (23.0%)	820 (25.8%)
Pack years	9.93 (14.10)	8.57 (13.45)	9.31 (13.82)
Diabetes	65 (3.8%)	81 (5.6%)	146 (4.6%)

*Note:* Study of Health in Pomerania (SHIP) Entries are means (standard deviations) for continuous variable and numbers of observations (%) for categorical variables.

### 2.2 Study Variables

Probing depth (PD) and clinical attachment levels (CAL) were measured using a manual periodontal probe (PCPUNC 15 Hu‐Friedy, Chicago, IL, USA) at four sites (distobuccal, midbuccal, mesiobuccal and midlingual/midpalatinal) in partial‐mouth examinations (left or right side, randomly selected at baseline and fixed for follow‐up) (Holtfreter et al. 2021). Third molars were excluded. PD was measured as the distance between the pocket base and the free gingival margin. CAL was measured as the distance between the cementoenamel junction and the pocket base. When identification of the cementoenamel junction was hindered by defect or restoration, the attachment level was not recorded. Measurements were mathematically rounded to the nearest millimetre. The Supplement gives detailed information about calibration data. Mean PD, the proportion of sites with PD ≥ 3 mm (pPD3), the proportion of sites with PD ≥ 4 mm (pPD4), mean CAL, the proportion of sites with CAL ≥ 3 mm (pCAL3) and the proportion of sites with CAL ≥ 4 mm (pCAL4) were calculated for each participant (Demmer et al. 2008). Participants were classified according to the Centers for Disease Control and Prevention/American Academy of Periodontology (CDC/AAP) case definition (Eke et al. 2012). We assessed average alcohol consumption using a validated beverage‐specific quantity‐frequency questionnaire covering the previous 30 days (Baumeister et al. 2018). Average alcohol consumption (ethanol in grams per day [g/day]) was calculated by multiplying frequency and amount of alcohol from beer, wine and spirits, respectively, using standard ethanol contents of 4.8% (by volume) in beer, 11% in wine and 33% in spirits for conversion. Confounders were selected using the modified disjunctive cause criterion (VanderWeele et al. 2021). Models were adjusted for covariates (age, sex, education, smoking status, pack years and diabetes) assessed at baseline (Table 1). Pack years was calculated as *a* × *b*/*c*, where *a* is the number of cigarettes smoked per day, *b* refers to the number of years of smoking and *c* refers to 20 cigarettes per pack. Diabetes was defined as self‐reported physician diagnosis or intake of antidiabetic medication. We further adjusted the models for follow‐up periodontal parameters (using dataset 2) for baseline values of the outcomes to minimise the risk of reverse causation (VanderWeele 2021; VanderWeele et al. 2016).

### 2.3 Statistical Analyses

Missing data were imputed using fully conditional specification, employing logistic regression for categorical variables (e.g., periodontitis) and predictive mean matching for continuous variables (White et al. 2011; Wijesuriya et al. 2025). Forty imputed datasets (m) were generated, following the guideline that the fraction of missing information divided by m should be ≤ 0.05 (White et al. 2011). Estimates and standard errors were combined using Rubin's rules (Morris et al. 2015). The association between baseline average alcohol consumption and incident periodontitis was assessed using a relative risk (RR) derived from multivariable Poisson regression. For mean PD and CAL at follow‐up, we used a generalised linear model with a gamma distribution and log link (Gamma‐GLM) to account for their skewed distributions (Manning et al. 2005). A fractional logit model was used to examine the relationship between alcohol consumption and pPD3, pPD4, pCAL3 and pCAL4, given that these are proportional outcomes (Wooldridge 2010). Because average alcohol consumption exhibited a semi‐continuous distribution, with a mass point at zero (non‐drinkers) and a heavy right tail (heavy drinkers), a two‐part approach was employed. Following recommendations in the literature (Lachin et al. 2020; Sauerbrei et al. 2020), we modelled alcohol intake using a binary variable (drinker vs. non‐drinker) and a continuous variable representing dose–response among drinkers. The continuous variable was centred by subtracting the mean alcohol intake among drinkers and modelled using a second‐degree fractional polynomial (Lorenz et al. 2017; Sauerbrei et al. 2020). To mitigate potential biases from residual confounding and reverse causation associated with using non‐drinkers as a reference group (e.g., the sick‐quitter effect), we used low‐volume drinking (≤ 10 g/day) as the comparison point (Im et al. 2023; Naimi and Chikritzhs 2025; Naimi et al. 2022; Rehm et al. 2008).

We tested whether dose–response associations differed between men and women using multiplicative interaction terms. We calculated the *E*‐value for residual confounding as well as the multibias *E*‐value to assess the sensitivity of the results to unmeasured confounders and selection bias simultaneously (Smith et al. 2021). These measures estimate the minimum value required for the sensitivity parameters of each bias to be consistent with a true null effect. Analyses were performed using R (version 4.4.2; R Foundation for Statistical Computing, Vienna, Austria) and Stata (version 18.5.; StataCorp, College Station, TX, USA). The reporting was based on recommendations by STROBE (STrengthening the Reporting of OBservational studies in Epidemiology).

## 3 Results

**FIGURE 1:**
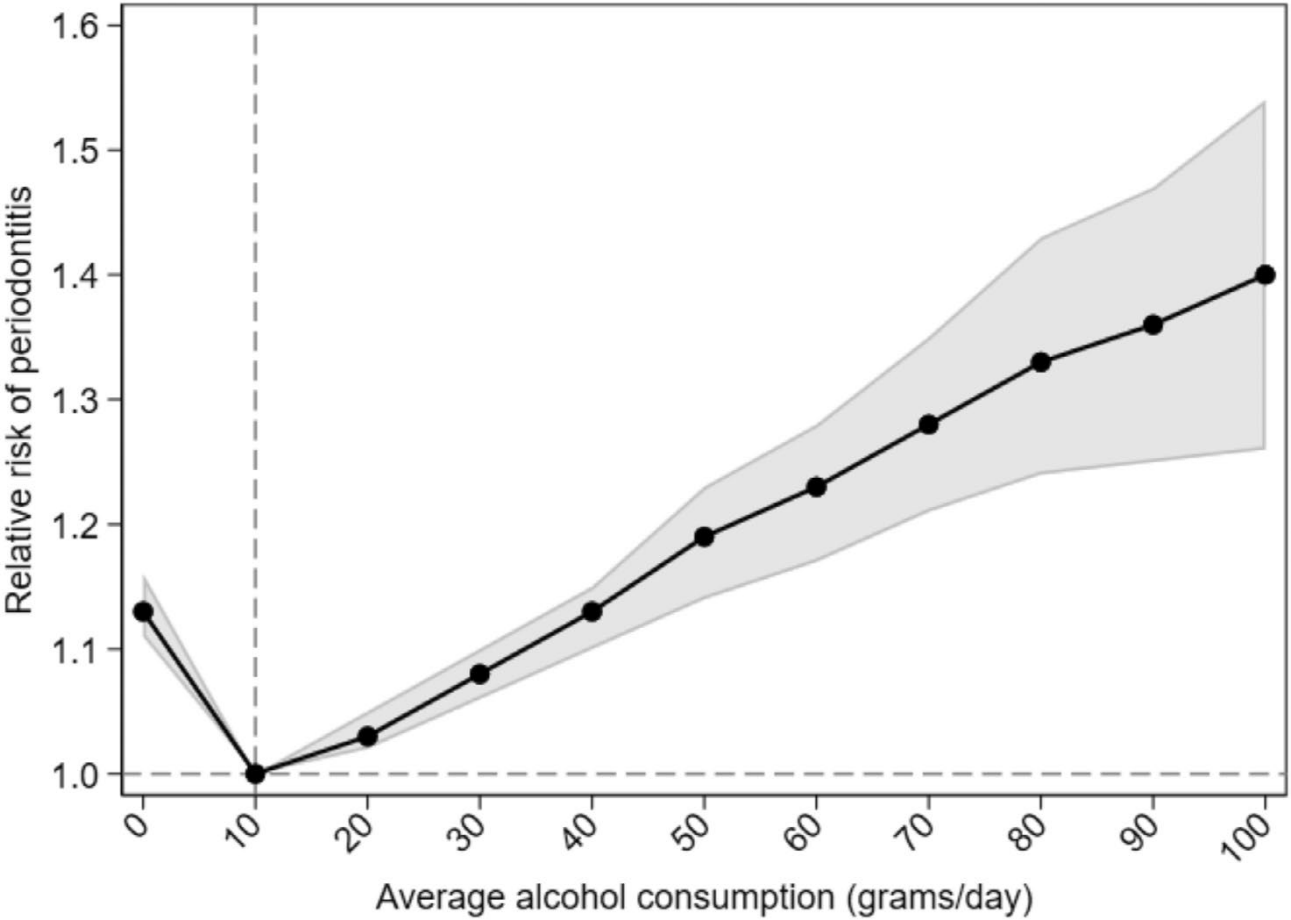
Association between baseline alcohol consumption and incident periodontitis. Multivariable Poisson regression model for incident moderate or severe periodontitis. Average alcohol consumption (grams ethanol per day) modelled using fractional polynomials with excess‐zero adjustment. Adjusted for age, sex, school education, smoking status, pack years and diabetes.

**FIGURE 2:**
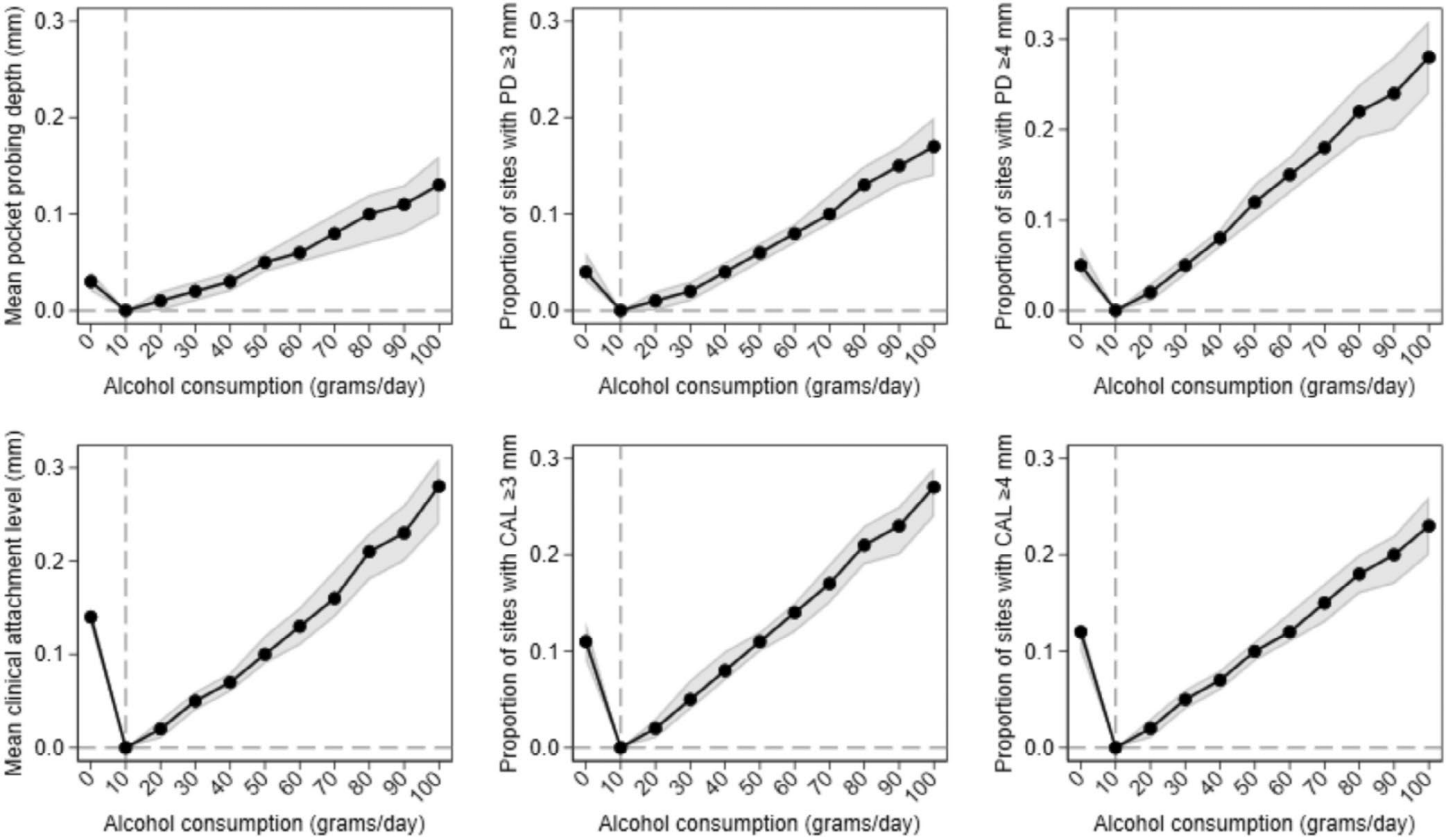
Association between baseline alcohol consumption and follow‐up periodontal parameters. Multivariable Gamma‐GLM for follow‐up PD and CAL. Fractional response logit model for follow‐up pPD3, pPD4, pCAL3 and pCAL4. Average alcohol consumption (grams ethanol per day) modelled using fractional polynomials with excess‐zero adjustment. Adjusted for baseline measure of the outcome variable, age, sex, school education, smoking status, packyears and diabetes.

In the dataset for examining the association between baseline average alcohol consumption and incident periodontitis, the mean age (standard deviation (SD)) in years was 44.1 (10.4) and 42% of the participants were men (Table 1). The mean average alcohol consumption (SD) was 10.6 (15.0) g/day. Over a median follow‐up of 5.0 years (IQR: 4.9–5.2), 282 out of 1282 participants developed moderate/severe periodontitis. Average alcohol intake at baseline was prospectively associated with incident moderate/severe periodontitis (Figure 1). The RRs were 1.08 (95% [confidence interval] CI: 1.06, 1.10) for 30 g/day and 1.23 (1.17, 1.28) for 60 g/day compared with 10 g/day. Analysis restricted to never‐smokers showed similar associations (RR of 1.10 [1.07, 1.12] for 30 vs. 10 g/day; RR of 1.29 (1.20, 1.39) for 60 vs. 10 g/day) (Figure S2). Baseline average alcohol consumption was also associated with periodontal parameters measured at follow‐up (Figure 2). We found no evidence of effect modification by sex. The confounding *E*‐values for RRs of 1.08 and 1.23 were 1.37 and 1.76, respectively. The multibias *E*‐values for RRs of 1.08 and 1.23 were 1.24 and 1.45.

## 4 Discussion

In this population‐based cohort study, we found an association between alcohol intake and periodontitis risk. Our findings advance the literature in three key ways. First, we modelled alcohol exposure with a non‐linear approach, distinguishing non‐drinkers from drinkers to better capture dose–response relationships. Second, to mitigate potential biases from sick‐quitting, confounding and misclassification, we used low‐level alcohol consumption, rather than abstinence, as the reference group (Naimi et al. 2022; Naimi and Chikritzhs 2025). Third, we quantified the robustness of our results to unmeasured confounding and selection bias through sensitivity analyses.

Our findings partially align with some, but not all, previous cohort studies examining the relationship between alcohol consumption and periodontitis. The largest cohort study, conducted among 39,461 male health professionals in the United States, found that intake of ≥ 30 g/day (compared with 0 g/day) at baseline was associated with an RR for periodontitis of 1.27 (1.08, 1.49) over a ~12‐year follow‐up (Pitiphat et al. 2003). The mean baseline age was 54 years. Alcohol was assessed using a beverage‐specific quantity‐frequency recall of 12 months at three measurements and modelled as a time‐varying covariate. At baseline, 24% were non‐drinkers, 24% consumed 0.1–4.9 g/day, 27% consumed 5–14.9 g/day, 13% consumed 15–29.9 g/day and 11% consumed ≥ 30 g/day. The study employed an extensive confounder adjustment set. However, updating confounder information without suitable modelling could have resulted in conditioning on indirect effects (Daniel et al. 2013). A prospective population study in Brazil assessed alcohol among 532 individuals and found a RR of 1.30 (1.07, 1.58) for CAL progression among those who consumed > 1 glass/day (Wagner et al. 2017). Alcohol consumption was assessed using a recall period of one week, and the average alcohol consumption was 2.9 g/day in the total study sample and 23 g/day among current drinkers. Strengths of the study are that it included young adults in the sample, the non‐linear modelling and adjustment for smoking history. These studies and ours contrast with two additional longitudinal studies. A study of 1332 Japanese men found no association between alcohol and periodontitis incidence over a 4‐year period (Okamoto et al. 2006). The mean age was 43.5 years, and alcohol was quantified as g/day. Among 30‐ to 39‐year‐old participants, 24% were non‐drinkers, 64% consumed 1–20 g/day and 12% consumed > 20 g/day. Among participants aged 40–49 years, 22% were non‐drinkers, 59% consumed 1–20 g/day and 19% consumed > 20 g/day. Among participants aged 50–59 years, 21% were non‐drinkers, 50% consumed 1–20 g/day and 29% consumed > 20 g/day. The study relied on data from individuals attending two consecutive dental check‐ups, and it is possible that those with higher alcohol consumption were underrepresented. It adjusted for relevant confounders but potentially over‐adjusted by defining smoking status using follow‐up information (Lu et al. 2021). Two Finnish studies examined the association between alcohol intake and the number of deep periodontal pockets at follow‐up. Sankaranarayanan et al. (2019) used data from the Health 2000 Survey and a follow‐up after 4 years (*n* = 244). Alcohol was measured using a beverage‐specific quantity‐frequency measure for the past 12 months and expressed as g/week, frequency and at‐risk consumption. Alcohol consumption at baseline was not reported. None of the alcohol variables were associated with the risk of deep periodontal pockets at follow‐up. Sankaranarayanan et al. (2020) used the Health 2000 Survey and its 10‐year follow‐up (Health 2011 Survey) (*n* = 362). Alcohol consumption was quantified as frequency of alcohol use, g/week and g/year. The means of g/week and g/year at baseline were 60.2 and 2752, respectively. No association was found between any of the alcohol exposure variables and the number of teeth with deep periodontal pockets at follow‐up. The study excluded participants with diabetes at baseline and adjusted for several confounding variables, including smoking and oral hygiene. However, it adjusted for covariates measured at follow‐up, which might have resulted in over‐adjustment. Both analyses based on the Health 2000 follow‐up were relatively small and may have lacked statistical power. Notably, previous studies used non‐drinkers as the reference group, potentially introducing sick‐quitter bias, selection bias, misclassification and confounding (Naimi et al. 2022; Naimi and Chikritzhs 2025; Oliveira et al. 2022). In a previous quasi‐experimental study, we used genetic variants as instrumental variables for long‐term alcohol exposure and found evidence for an increase in periodontitis risk (Baumeister et al. 2021). Although this genetic approach may be subject to its own bias, its immunity to behavioural and social confounders provides independent support for the effect of alcohol on periodontitis.

The postulated mechanisms linking alcohol intake with periodontitis include impaired host defence (impaired neutrophil, macrophage and T‐cell function), altered cytokines and tumour necrosis factor‐α levels (Barr et al. 2016; Szabo and Saha 2015), contributing to bacterial proliferation and penetration. Moreover, individuals chronically exposed to high alcohol intake show differences in subgingival microbial composition and have higher proportions of periodontal pathogens (Oliveira et al. 2023). High alcohol consumption may also affect periodontitis through poor oral hygiene habits and reduced dental care attendance (Oliveira et al. 2022).

Several limitations warrant consideration. A major concern is uncontrolled bias that could distort risk estimates. We have no reason to believe that misclassification of periodontitis was differential by exposure level, and non‐differential outcome misclassification would, on average, bias towards the null in this situation (Copeland et al. 1977). However, residual confounding from imprecisely measured smoking history and unmeasured oral hygiene practices could explain our observed associations. Selection bias may have occurred if study participation varied by drinking status. Our single baseline alcohol measurement with 30‐day recall could have led to regression dilution bias, suggesting the need for repeated assessments beginning in younger populations. Partial‐mouth periodontal assessments (vs. full‐mouth protocols) may attenuate or inflate associations (Alshihayb et al. 2020). Furthermore, our study lacked an outcome measure reflecting the amount of inflamed periodontal tissue. Finally, the exclusively Caucasian study population limits the generalisability of our findings.

In conclusion, this study indicates that alcohol consumption increases periodontitis risk, although potential confounding and selection bias could have biased our results. Our findings highlight the need for further research to elucidate the relationship between alcohol consumption and periodontitis. Future prospective cohort studies should prioritise repeated, detailed assessments of alcohol intake, ideally beginning in adolescence, to capture the full cumulative alcohol exposure over time. Critically, these studies must also incorporate comprehensive and repeated measurements of potential confounders, including smoking, oral hygiene and socio‐economic status, and employ appropriate statistical methods to adjust for their influence. Furthermore, quasi‐experimental study designs and rigorous evaluations of policies aimed at reducing population alcohol consumption are essential to establish a stronger causal link and provide a more robust evidence base for public health recommendations and policy decisions. Such research will be crucial for developing effective strategies to mitigate the adverse oral health effects associated with alcohol consumption.

## Author Contributions

S.‐E.B. contributed to conception, design, data acquisition, performed the analysis and drafted the manuscript. J.S., G.G.N., H.V., T.K. and B.H. contributed to data acquisition and interpretation, and critically revised the manuscript. All authors contributed to manuscript revision, and read and approved the submitted version.

## Conflicts of Interest

The authors declare no conflicts of interest.

## Supporting information


Data S1.

## Data Availability

The data that support the findings of this study are available on request from the corresponding author. The data are not publicly available due to privacy or ethical restrictions.
