# Supplementary material for: Alcohol Consumption, Risk of Periodontitis and Change of Periodontal Parameters in a Population‐Based Cohort Study
Source: J Clin Periodontol. 2025 Mar 13;52(7):1024–31. doi: 10.1111/jcpe.14154 (PMC12176462; doi:10.1111/jcpe.14154)
Supplement: Supplementary file 1 — Data S1. [file JCPE-52-1024-s001.docx]

# Alcohol consumption, risk of periodontitis and change of periodontal parameters in a population-based cohort study

Supplement

# Methods

## Calibration data

In SHIP-0, dental examinations were conducted by eight calibrated and licensed dentists. Biannually, calibration exercises were performed on test patients not connected to the study, yielding intra-rater correlations between 0.68 and 0.88 and an inter-rater correlation of 0.72 for PD and intra-rater correlations of 0.82–0.91 per examiner and an inter-rater correlation of 0.84 for CAL. In SHIP-1, intra-rater correlations for CAL were 0.70 to 0.89, while the interrater correlation was 0.85. For PD, intra-rater correlations were 0.43 to 0.82, and the interrater correlation was 0.78. In SHIP-TREND-0, dental examinations were conducted by five calibrated dentists. In calibration exercises, all dentists repeatedly examined five people who were not connected to the study. Intra-rater correlations for CAL measurements ranged between 0.67 and 0.89 and inter-rater correlation was 0.70. For PD measurements, the examiners yielded intra-rater correlations between 0.68 and 0.88 and an inter-rater correlation of 0.72. For assessment of the tooth status, Cohen’s kappa reliability coefficients were 0.93-0.99 (intra-examiner) and 0.94-0.98 (pairwise inter-examiner). In SHIP-TREND-1, dental examinations were conducted by four calibrated dentists. In calibration exercises, all dentists repeatedly examined five people who were not connected to the study. Intra-rater correlations for CAL measurements ranged between 0.90 and 0.96 and pairwise inter-rater correlations of 0.86-0.94. For PD measurements, the examiners yielded intra-rater correlations between 0.77 and 0.91 and pairwise inter-rater correlations of 0.63-0.85. For assessment of the tooth status, Cohen’s kappa reliability coefficients were 0.97-1.00 (intra-examiner) and 0.91-0.96 (pairwise inter-examiner).

Supplementary Figure 1 Flow-chart of study population
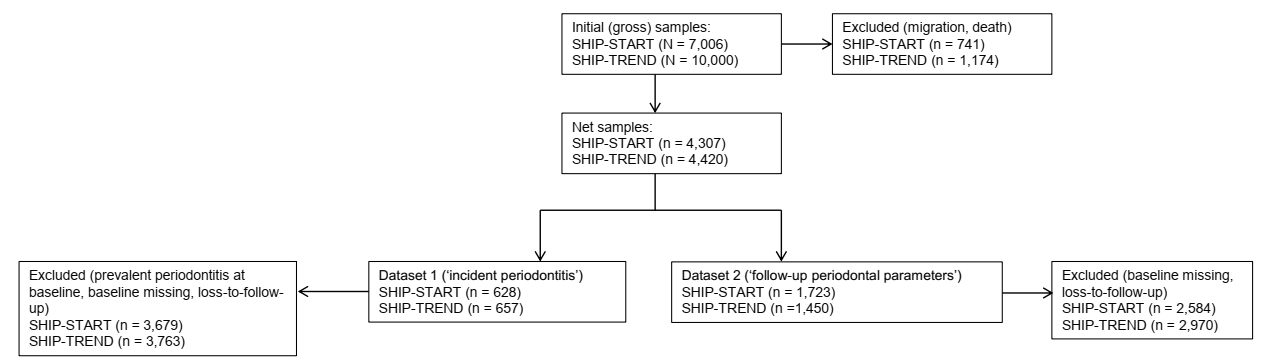


Supplementary Figure 2 Association between baseline alcohol consumption and incident periodontitis in never-smokers


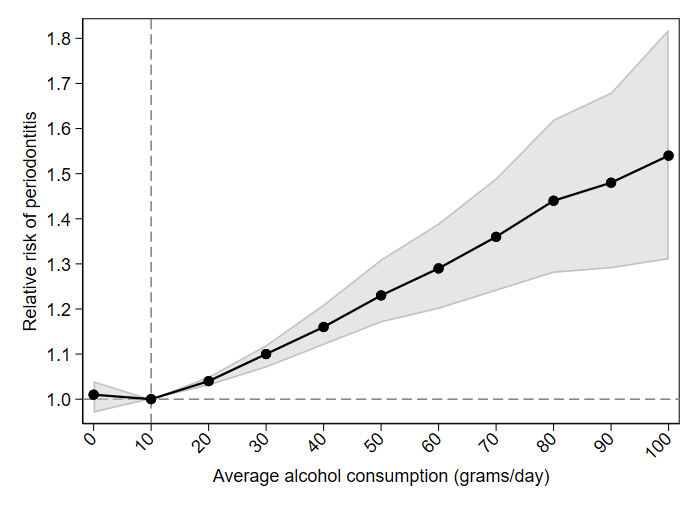
Multivariable Poisson regression model for incident moderate or severe periodontitis. Average alcohol consumption (grams ethanol per day) modeled using fractional polynomials with excess-zero adjustment. Adjusted for age, sex, school education, and diabetes.

Supplementary Table 1 Baseline descriptive statistics of analytical dataset 1 to examine the association between average alcohol consumption and risk of incident periodontitis in SHIP-START and SHIP-TREND without listwise deletion of baseline missing values

|  | SHIP-START-0 | SHIP-TREND-0 | Total |
| --- | --- | --- | --- |
| Sample, n | 2,381 (47.0%) | 2,688 (53.0%) | 5,069 (100.0%) |
| Periodontitis |  |  |  |
| No or mild | 1,537 (100.0%) | 1,616 (100.0%) | 3,153 (100.0%) |
| Moderate or severe | 0 (0%) | 0 (0%) | 0 (0%) |
| Missing values | 844 (35.5%) | 1,072 (39.9%) | 1,916 (37.8%) |
| Mean pocket probing depth, mm | 2.23 (0.67) | 2.33 (0.59) | 2.28 (0.63) |
| Missing values | 567 (23.8%) | 798 (29.7%) | 1,365 (26.9%) |
| Proportion of sites with pocket probing depth ≥3mm | 0.35 (0.23) | 0.34 (0.21) | 0.35 (0.22) |
| Missing values | 570 (23.9%) | 798 (29.7%) | 1,368 (27.0%) |
| Proportion of sites with pocket probing depth ≥4mm | 0.06 (0.15) | 0.07 (0.15) | 0.06 (0.15) |
| Missing values | 570 (23.9%) | 798 (29.7%) | 1,368 (27.0%) |
| Mean clinical attachment level, mm | 1.51 (1.53) | 1.48 (1.21) | 1.50 (1.37) |
| Missing values | 741 (31.1%) | 987 (36.7%) | 1,728 (34.1%) |
| Proportion of sites with clinical attachment level ≥3mm | 0.22 (0.27) | 0.16 (0.23) | 0.19 (0.25) |
| Missing values | 743 (31.2%) | 987 (36.7%) | 1,730 (34.1%) |
| Proportion of sites with clinical attachment level ≥4mm | 0.08 (0.21) | 0.06 (0.18) | 0.07 (0.19) |
| Missing values | 743 (31.2%) | 987 (36.7%) | 1,730 (34.1%) |
| Average alcohol consumption, grams ethanol per day | 10.91 (17.52) | 7.40 (12.06) | 9.00 (14.90) |
| Missing values | 167 (7.0%) | 33 (1.2%) | 200 (3.9%) |
| Male sex | 1,079 (45.3%) | 1,192 (44.3%) | 2,271 (44.8%) |
| Missing values | 0 (0%) | 0 (0%) | 0 (0%) |
| Age, years | 47.40 (18.35) | 49.60 (16.60) | 48.57 (17.48) |
| Missing values | 0 (0%) | 0 (0%) | 0 (0%) |
| Schooling attainment |  |  |  |
| <10 years | 862 (36.4%) | 620 (23.1%) | 1,482 (29.4%) |
| 10 years | 1,071 (45.2%) | 1,344 (50.1%) | 2,415 (47.9%) |
| >10 years | 434 (18.3%) | 716 (26.7%) | 1,150 (22.8%) |
| Missing values | 14 (0.6%) | 8 (0.3%) | 22 (0.4%) |
| Smoking status |  |  |  |
| Never smoker | 872 (36.8%) | 1,000 (37.4%) | 1,872 (37.1%) |
| Former smoker | 779 (32.9%) | 953 (35.7%) | 1,732 (34.4%) |
| Current smoker | 719 (30.3%) | 718 (26.9%) | 1,437 (28.5%) |
| Missing values | 11 (0.6%) | 17 (0.6%) | 28 (0.6%) |
| Pack years | 8.95 (13.45) | 9.59 (14.84) | 9.28 (14.19) |
| Missing values | 410 (17.2%) | 556 (20.7%) | 966 (19.1%) |
| Diabetes | 139 (5.8%) | 274 (10.2%) | 413 (8.2%) |
| Missing values | 0 (0%) | 8 (0.3%) | 8 (0.2%) |

SHIP: Study of Health in Pomerania Entries are means (standard deviations) for continuous variable and numbers of observations (%) for categorical variables.

Supplementary Table 2 Baseline descriptive statistics of analytical dataset 2 to examine the association between average alcohol consumption and follow-up periodontal parameters in the Study of Health in Pomerania START and TREND without listwise deletion of missing values

|  | SHIP-START-0 | SHIP-TREND-0 | Total |
| --- | --- | --- | --- |
| Sample, n | 4,307 (49.4%) | 4,420 (50.6%) | 8,727 (100.0%) |
| Periodontitis |  |  |  |
| No or mild | 1,537 (44.4%) | 1,616 (48.3%) | 3,153 (46.3%) |
| Moderate or severe | 1,926 (55.6%) | 1,732 (51.7%) | 3,658 (53.7%) |
| Missing values | 844 (19.6%) | 1,072 (24.3%) | 1,916 (22.0%) |
| Mean pocket probing depth, mm | 2.53 (0.76) | 2.59 (0.71) | 2.56 (0.73) |
| Missing values | 567 (13.2%) | 798 (18.1%) | 1,365 (15.6%) |
| Proportion of sites with pocket probing depth ≥3mm | 0.46 (0.25) | 0.43 (0.23) | 0.45 (0.24) |
| Missing values | 573 (13.3%) | 798 (18.1%) | 1,371 (15.7%) |
| Proportion of sites with pocket probing depth ≥4mm | 0.13 (0.18) | 0.14 (0.19) | 0.14 (0.19) |
| Missing values | 573 (13.3%) | 798 (18.1%) | 1,371 (15.7%) |
| Mean clinical attachment level, mm | 2.63 (1.89) | 2.47 (1.69) | 2.55 (1.80) |
| Missing values | 741 (17.2%) | 987 (22.3%) | 1,728 (19.8%) |
| Proportion of sites with clinical attachment level ≥3mm | 0.47 (0.35) | 0.40 (0.35) | 0.43 (0.35) |
| Missing values | 746 (17.3%) | 987 (22.3%) | 1,733 (19.9%) |
| Proportion of sites with clinical attachment level ≥4mm | 0.28 (0.32) | 0.25 (0.31) | 0.26 (0.32) |
| Missing values | 746 (17.3%) | 987 (22.3%) | 1,733 (19.9%) |
| Average alcohol consumption, grams ethanol per day | 11.75 (18.28) | 8.45 (13.43) | 10.02 (16.01) |
| Missing values | 315 (7.3%) | 51 (1.1%) | 366 (4.2%) |
| Male sex | 2,115 (49.1%) | 2,145 (48.5%) | 4,260 (48.8%) |
| Missing values | 0 (0%) | 0 (0%) | 0 (0%) |
| Age, years | 49.81 (16.39) | 51.96 (15.46) | 50.90 (15.96) |
| Missing values | 0 (0%) | 0 (0%) | 0 (0%) |
| Schooling attainment |  |  |  |
| <10 years | 1,714 (40.1%) | 1,029 (23.3%) | 2,743 (31.6%) |
| 10 years | 1,864 (43.6%) | 2,268 (51.5%) | 4,132 (47.6%) |
| >10 years | 699 (16.3%) | 1,110 (25.2%) | 1,809 (20.8%) |
| Missing values | 30 (0.7%) | 13 (0.3%) | 43 (0.5%) |
| Smoking status |  |  |  |
| Never smoker | 1,536 (35.8%) | 1,605 (36.5%) | 3,141 (36.2%) |
| Former smoker | 1,453 (33.9%) | 1,610 (36.6%) | 3,063 (35.3%) |
| Current smoker | 1,299 (30.3%) | 1,183 (26.9%) | 2,482 (28.6%) |
| Missing values | 19 (0.7%) | 22 (0.5%) | 41 (0.5%) |
| Pack years | 10.54 (15.04) | 10.70 (15.61) | 10.62 (15.32) |
| Missing values | 679 (15.8%) | 877 (19.8) | 1,556 (17.8%) |
| Diabetes | 265 (6.2%) | 461 (10.5%) | 726 (8.3%) |
| Missing values | 0 (0%) | 11 (0.2%) | 11 (0.1%) |

Entries are means (standard deviations) for continuous variable and numbers of observations (%) for categorical variables.
